# A Prosaposin-Derived Peptide Alleviates Kainic Acid-Induced Brain Injury

**DOI:** 10.1371/journal.pone.0126856

**Published:** 2015-05-18

**Authors:** Hiroaki Nabeka, Tetsuya Shimokawa, Takuya Doihara, Shouichiro Saito, Hiroyuki Wakisaka, Fumihiko Hamada, Naoto Kobayashi, Seiji Matsuda

**Affiliations:** 1 Department of Anatomy and Embryology, Ehime University Graduate School of Medicine, Toon, Ehime, Japan; 2 Laboratory of Veterinary Anatomy, Faculty of Applied Biological Sciences, Gifu University, Yanagido, Gifu, Japan; 3 Ehime Prefectural University of Health Sciences, Iyo, Ehime, Japan; 4 Department of Human Anatomy, Oita University Fuculty of Medicine, Yufu, Oita, Japan; 5 Medical Education Center, Ehime University Graduate School of Medicine, Toon, Ehime, Japan; St Michael's Hospital, University of Toronto, CANADA

## Abstract

Four sphingolipid activator proteins (i.e., saposins A–D) are synthesized from a single precursor protein, prosaposin (PS), which exerts exogenous neurotrophic effects in vivo and in vitro. Kainic acid (KA) injection in rodents is a good model in which to study neurotrophic factor elevation; PS and its mRNA are increased in neurons and the choroid plexus in this animal model. An 18-mer peptide (LSELIINNATEELLIKGL; PS18) derived from the PS neurotrophic region prevents neuronal damage after ischemia, and PS18 is a potent candidate molecule for use in alleviating ischemia-induced learning disabilities and neuronal loss. KA is a glutamate analog that stimulates excitatory neurotransmitter release and induces ischemia-like neuronal degeneration; it has been used to define mechanisms involved in neurodegeneration and neuroprotection. In the present study, we demonstrate that a subcutaneous injection of 0.2 and 2.0 mg/kg PS18 significantly improved behavioral deficits of Wistar rats (*n* = 6 per group), and enhanced the survival of hippocampal and cortical neurons against neurotoxicity induced by 12 mg/kg KA compared with control animals. PS18 significantly protected hippocampal synapses against KA-induced destruction. To evaluate the extent of PS18- and KA-induced effects in these hippocampal regions, we performed histological evaluations using semithin sections stained with toluidine blue, as well as ordinal sections stained with hematoxylin and eosin. We revealed a distinctive feature of KA-induced brain injury, which reportedly mimics ischemia, but affects a much wider area than ischemia-induced injury: KA induced neuronal degeneration not only in the CA1 region, where neurons degenerate following ischemia, but also in the CA2, CA3, and CA4 hippocampal regions.

## Introduction

**Fig 1:**
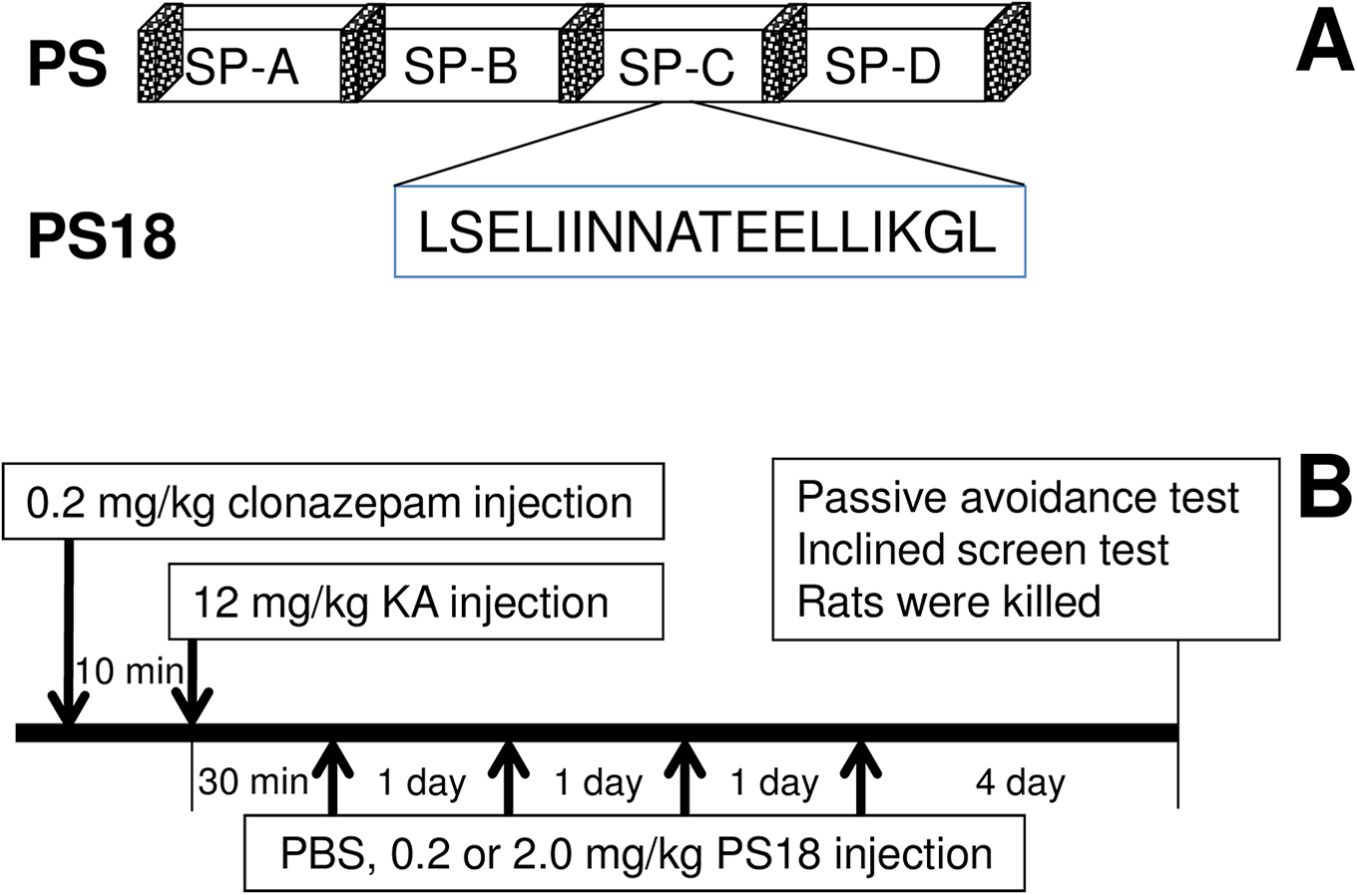
(A) The structure of prosaposin (PS) and PS18. PS contains four saposins, and saposin-C contains the neurotrophic sequence, PS18. (**B**) Experimental design of kainic acid (KA) and PS18 injections administered to rats. The rats were randomly allocated into four groups, and received a subcutaneous injection of clonazepam (0.2 mg/kg), followed 10 minutes later by subcutaneous injections of KA (12 mg/kg body weight) and PS18 or PBS once a day for 3 consecutive days. The control animals were injected with PBS under the same regimen, and all animals were killed after the passive avoidance and inclined screen tests, 7 days after the KA injection.

Prosaposin (PS) is a precursor protein for four small lysosomal glycoproteins: saposins A–D (Fig 1A). Each saposin activates specific lysosomal sphingolipid hydrolases, including cerebrosidase, ceramidase, sphingomyelinase, galactosidase, and arylsulfatase [1–4]. Both PS and the saposins are widely expressed in various tissues [5], although the brain, skeletal muscle, and heart cells predominantly contain unprocessed PS rather than saposins [6–11]. In addition, unprocessed PS is found in various secretory fluids, such as seminal plasma, bile, pancreatic juice, human breast milk, and cerebrospinal fluid [12,13]. PS mRNA and PS are strongly expressed in the choroid plexus [14,15].

PS is ubiquitously expressed in nervous tissues [9,16] and has been identified as a potent neurotrophic factor in addition to its role as a saposin precursor [17]. PS and a PS-derived peptide contain a neurotrophic activity domain, promote neurite outgrowth in neuroblastoma cells [17], and prevent programmed cell death in both cultured cerebral granule neurons [18,19] and cultured glial cells [20,21]. We reported that PS and PS18 (Fig 1A) facilitated sciatic nerve regeneration [22] and rescued both ischemic hippocampal CA1 neurons [23,24] and Corti’s organ by inducing expression of the anti-apoptotic molecule B cell lymphoma (Bcl)-2 [25]. PS and PS18 also rescued dopaminergic neurons from 1-methyl-4-phenyl-1,2,3,6-tetrahydropyridine (MPTP)-induced neurotoxicity via the upregulation of Bcl-2 or by inhibition of c-Jun, Bcl-2-like protein 4 (BAX), and caspase-3 [26].

Kainic acid (KA), a glutamate analog, is a powerful neurotoxic agent [27] that stimulates excitatory neurotransmitter release [28], and a systemic KA injection induces neuronal degeneration in certain brain areas, including the piriform cortex, amygdaloid complex, hippocampus, and septum [29–33]. The nature of neuronal degeneration caused by a systemic KA injection resembles some forms of ischemia [34].

Although PS receptors have been defined after debate over the past two decades [35], the nature of PS movement in injured and normal nervous tissue is unclear. We showed that PS and its mRNA increase in the facial nerve nucleus after nerve transection [36,37] and decrease in the brain of mdx (X chromosome-linked muscular dystrophy) mice [38]. In a previous study, we showed an increase in PS and PS mRNA in brain neurons and in the choroid plexus after a systemic KA injection [15].

In the present study, we investigated the neurotrophic effects of four subcutaneous PS18 injections following KA administration in rats. We evaluated the effects using a conventional step-down passive avoidance test and an inclined screen test, and by counting the number of intact neurons and synapses in the hippocampus and in the cerebral cortex. Based on these findings, PS18 efficacy and mechanism of action for the treatment of KA-induced neuronal damage and learning disability are discussed.

## Materials and Methods

### Animals

Ten-week-old male Wistar rats (CLEA, Japan) (*n* = 6 per group) were used in this study. All animals were housed at a constant temperature (22°C) under a 12:12 hour light: dark cycle and given food and water *ad libitum*. The experiments were conducted in accordance with ARRIVE guidelines and the Guide for Animal Experimentation of the Ehime University School of Medicine, Japan. The protocol was approved by the Animal Care Committee of Ehime University (Permit Number: 05A261).

### KA Administration (Fig 1B)

Rats were anesthetized with diethyl ether, and clonazepam (an anticonvulsant) was intraperitoneally (i.p.) injected (0.2 mg/kg). After 10 minutes, rats were anesthetized again with diethyl ether, and 12 mg/kg of KA, dissolved in phosphate-buffered saline (PBS), was subcutaneously injected [32]. After KA injection, the animals were housed at a constant temperature (22°C), as KA effects are at least partly dependent on body temperature [15]. The dose of KA was chosen based on the results of previous studies [15], and clonazepam did not result in any obvious convulsion during the experimental time, and all 24 rats were survived until the fixation.

### Administration of PS-Derived Artificial Peptide (Fig 1B)

Based on the findings of Kotani *et al*. [24] and Gao *et al*. [38], an 18-mer artificial peptide comprising the rat PS neurotrophic sequence (LSELIINNATEELLIKGL; PS 18; Fig 1A) was prepared by a commercial protein synthesis service (Operon, Tokyo, Japan). Animals were randomly allocated into four groups, with three KA exposure groups: (1) six animals were treated with 0.2 mg/kg PS18 in PBS, (2) six animals were treated with 2.0 mg/kg PS18, and (3) six animals were treated with the same volume of PBS. These doses of PS18 were used because they have previously been shown to result in some amount of neuronal damage [25,26]. PS18 was subcutaneously injected four times: immediately, and 1, 2, and 3 days after the KA injection. Six control rats were exposed to the same procedures as KA-treated animals, without KA or PS treatment.

### Step-Down Passive Avoidance Test

Seven days after the KA injection, the rats were examined with a conventional step-down passive avoidance apparatus that was divided into a safe platform and a grid floor. The safe platform was made of acrylic fiber with a floor constructed of stainless-steel grids. A scrambled DC constant current shock generator (Neuroscience Inc., Tokyo, Japan) delivered a 0.35 mA shock through the grid. The safe platform was also made of acryl fibers and was fixed to one side of the apparatus. Passive avoidance training was performed 7 days after the KA injection (Fig 1B). Each animal was initially placed on the safe platform. When the rat stepped down onto the grid floor, it received a foot shock. Although the rats initially repeatedly traveled up and down between the platform and the grid floor, they eventually remained on the platform. This training session lasted 300 seconds. Twenty-four hours later, each rat was again placed on the safe platform while the shock generator was turned off, and the response latency was measured. This test session also lasted 300 seconds [39,40].

### Inclined Screen Test

To assess the muscle strength of all four legs, the rats were placed on a horizontal screen. When the screen was gradually rotated into a vertical position, the rat gripped the screen and eventually fell. The degree at which the animal fell from the screen was recorded. This test was conducted 7 days after the KA injection following the passive avoidance test (Fig 1B). Each test included six trials in a single day.

### Counting of Neurons and Synapses

Hippocampal CA1 has been used to evaluate neuronal damage and trophic effects of drugs or factors after ischemia [39,40], since CA1 pyramidal neurons are particularly vulnerable to ischemic damage, and neurons compactly lining the stratum pyramidale are easy to count. Seven days after the KA injection, and following the passive avoidance and inclined screen tests, each animal was anesthetized by i.p. injection of chloral hydrate (10 mg/kg) and transcardially perfused, first with 50 mL PBS, and then with 300 mL 4% paraformaldehyde (PFA) in 0.1 M phosphate buffer (PB). Coronal slices (1,000 *μ*m) were cut 3.0–4.0 mm posterior to bregma using brain slicing equipment (Activational Systems, Inc., Warren, MI, USA) and used for electron microscopy. The brain blocks that were sectioned 4.0 mm posterior to bregma were used for light microscopy. The blocks were post-fixed in the same solution for 4 hours and embedded in paraffin using conventional methods, sectioned around the level 4.0 mm posterior to bregma, deparaffinized, and stained with hematoxylin and eosin (H&E).

For electron microscopy or semithin sections, the 1,000-*μ*m slices (3.0–4.0 mm posterior to bregma) were post-fixed in 3% glutaraldehyde in 0.1 M PB, post-fixed in 4% osmium tetroxide, block-stained with uranyl acetate, and embedded in epoxy resin. Semithin sections around the level 3.9 mm posterior to bregma were cut with a glass or sapphire knife at a 0.5 *μ*m thickness and stained with toluidine blue for light microscopy. Ultrathin sections were cut with a diamond knife, mounted on single-slot grids, and dual stained with uranyl acetate and lead citrate for electron microscopy.

**Fig 2:**
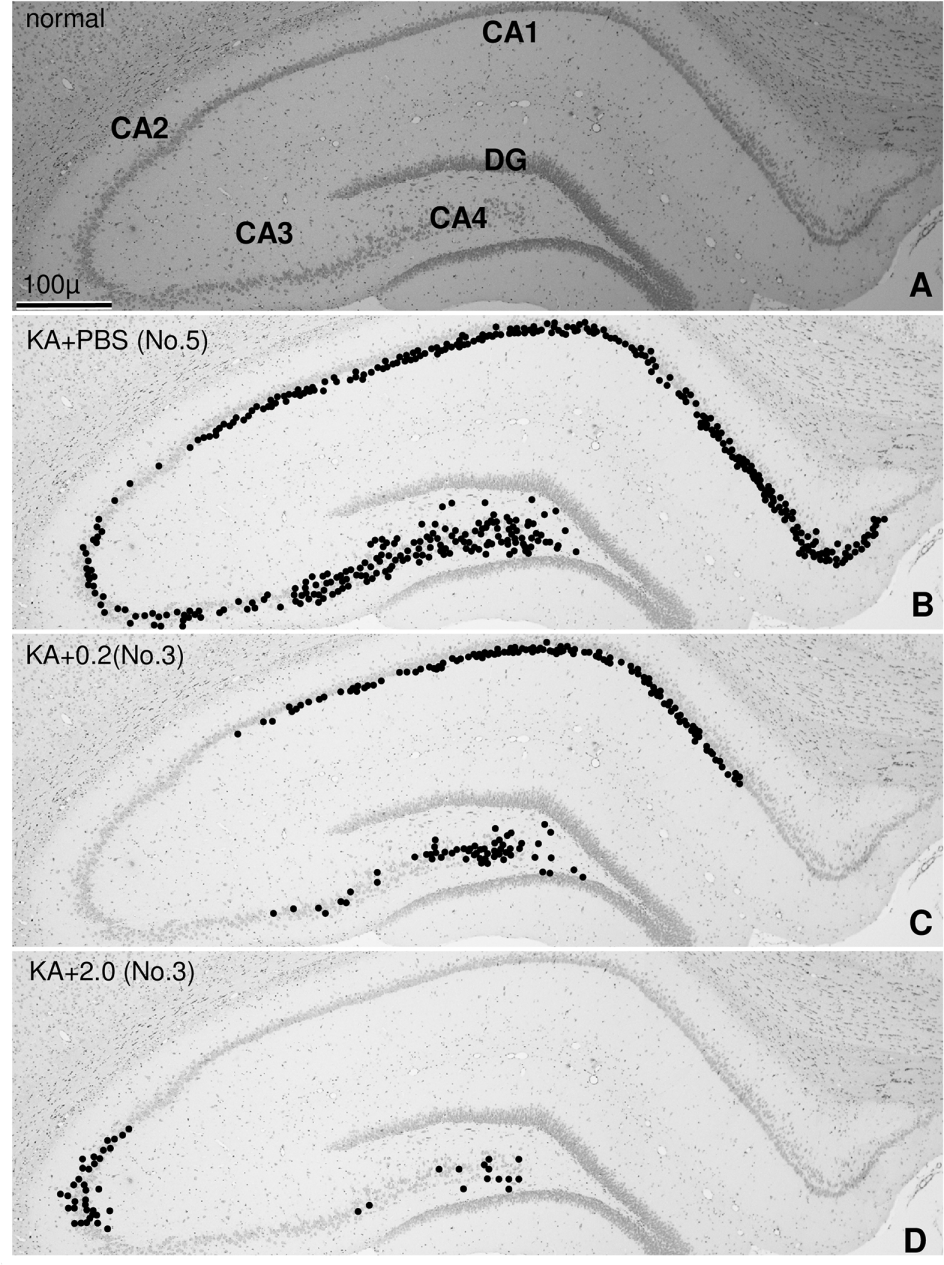
Histological Examination of KA-Induced Brain Injury. Plotting of injured neurons in the hippocampus of a normal control rat (A) and rats that received injections of PBS (B), 0.2 mg/kg PS18 (C), or 2.0 mg/kg PS18 after a KA injection (D). Each dot indicates one injured neuron. More than half of the neurons in the CA1, CA3 and CA4 regions were damaged in the severely damaged hippocampus (B), more than half of the neurons in the CA1 region were damaged in the moderately damaged hippocampus (C), and fewer than half of the neurons in the CA3 and CA4 regions were damaged in the weakly damaged hippocampus (D). Note that neuronal damage in CA3 was observed in (C) but not in (B). Scale bar = 100 μm.

We roughly classified the overall hippocampal damage into three groups: severe moderate, and weak injury. Severe injury was defined as damage to more than half of the neurons in the CA1, CA3 and CA4 hippocampal regions (Fig 2B), moderate as damage to more than half the neurons in the CA1 (Fig 2C), and weak injury as damage to fewer than half the neurons in the CA3 and CA4 regions (Fig 2D). This classification system is arbitrary, and is further described in the discussion.

We focused on a 1 mm linear length of hippocampal CA1, CA3, and CA4, and a 0.25 mm linear length of the dentate gyrus (DG). In toluidine blue-stained sections of these areas, pyramidal neurons with an intact morphological appearance or with a darkly stained injured appearance were counted. Neurons were counted in 0.5 × 2 mm, 0.25 × 4 mm, 0.5 × 2 mm, and 0.25 × 1 mm sections in CA1, CA3, CA4, and DG, respectively.

Electron micrographs of the central area (280 *μ*m^2^) of each CA1 stratum were acquired, and intact synapses with thick, apposed membranes and synaptic vesicles in the area were counted [39–40].

**Fig 3:**
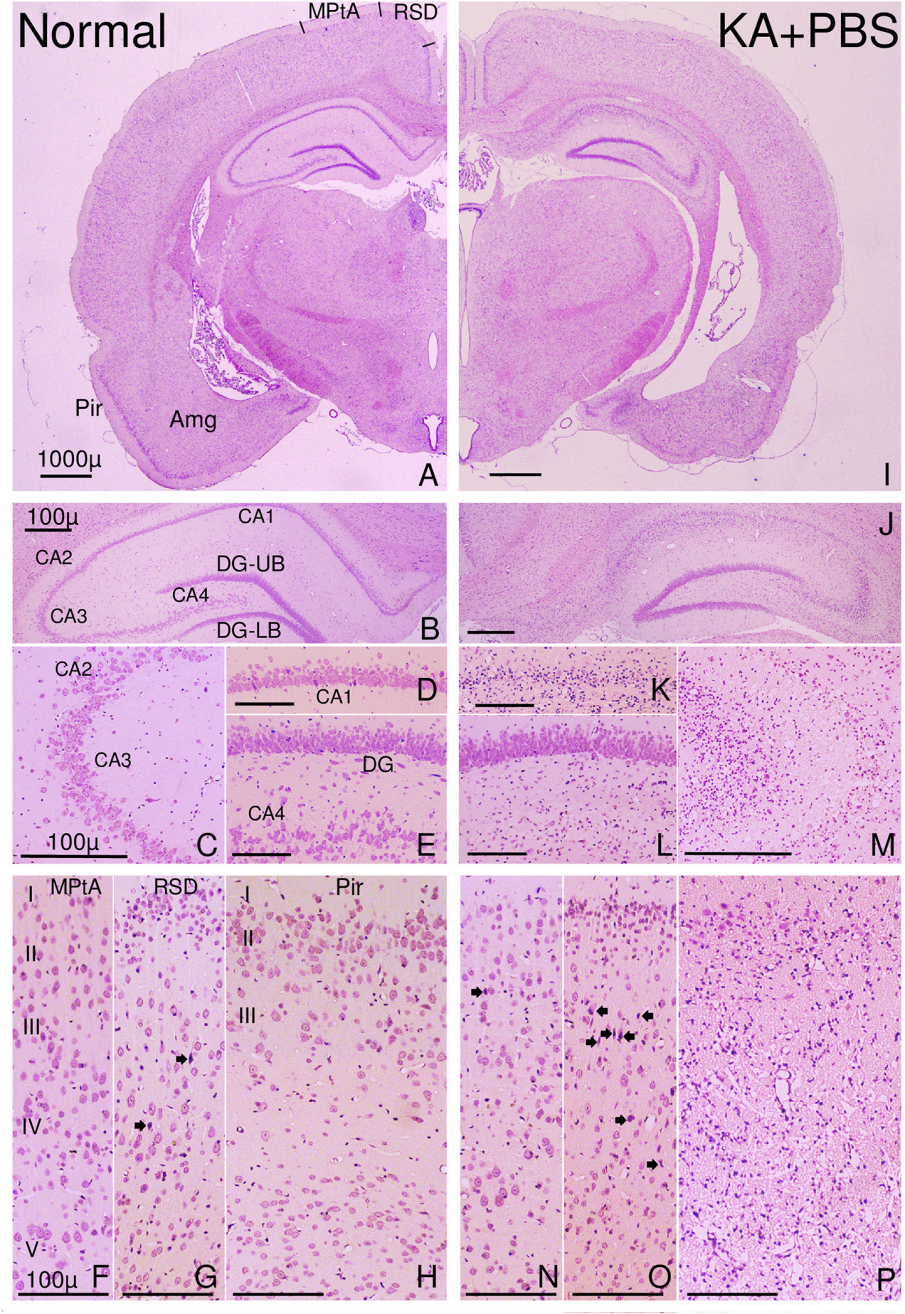
Photomicrographs of the forebrain of a normal rat (left half) or a KA and PBS-injected rat (right half) with severe damage in the hippocampus and piriform (Pir) cortex. Many neurons in CA1-4 were damaged in the hippocampus of the rat injected with KA (J–M). In the cortex of the rat injected with KA (N–P), few neurons were damaged in the medial parietal association cortex (MPtA), some neurons were damaged in the retrosplenial dysgranular cortex (RSD; O), and many neurons were damaged in the piriform cortex (P). Damaged neurons are indicated by closed arrows. Also, some dead neurons were detected in the RSD of normal rats (G). DG-UB, DG-LB: Upper and lower blade of the dentate gyrus (DG). Scale bar = 1,000 μm (A, I); scale bar = 100 μm (B–H, J–P).

The cerebral cortex was also carefully examined in the H&E sections (Fig 3). The six layers of the center of the occipital cortex (Fig 3F and 3N), retrosplenial granular cortex (Fig 3G and 3O), and three layers of the piriform cortex (Fig 3H and 3P) at the level 4.0 mm posterior to bregma are shown. Neurons with an intact morphological appearance in a 0.5 mm linear length of piriform cortex layer II were counted in two sections (Fig 3H and 3P).

### Statistical Analysis of Response Latencies and the Number of Neurons and Synapses

All experiments were done blindly (with respect to experimental groups) before the passive avoidance and inclined screen tests, and before neuron and synapse counting. The statistical significance of the effect of PS18 was examined by a one-way analysis of variance (ANOVA) and Fisher’s PLSD *post hoc* tests using the program StatView (Abacus Concepts Inc., Berkeley, CA, USA).

## Results

### Passive Avoidance and Inclined Screen Tests

**Fig 4:**
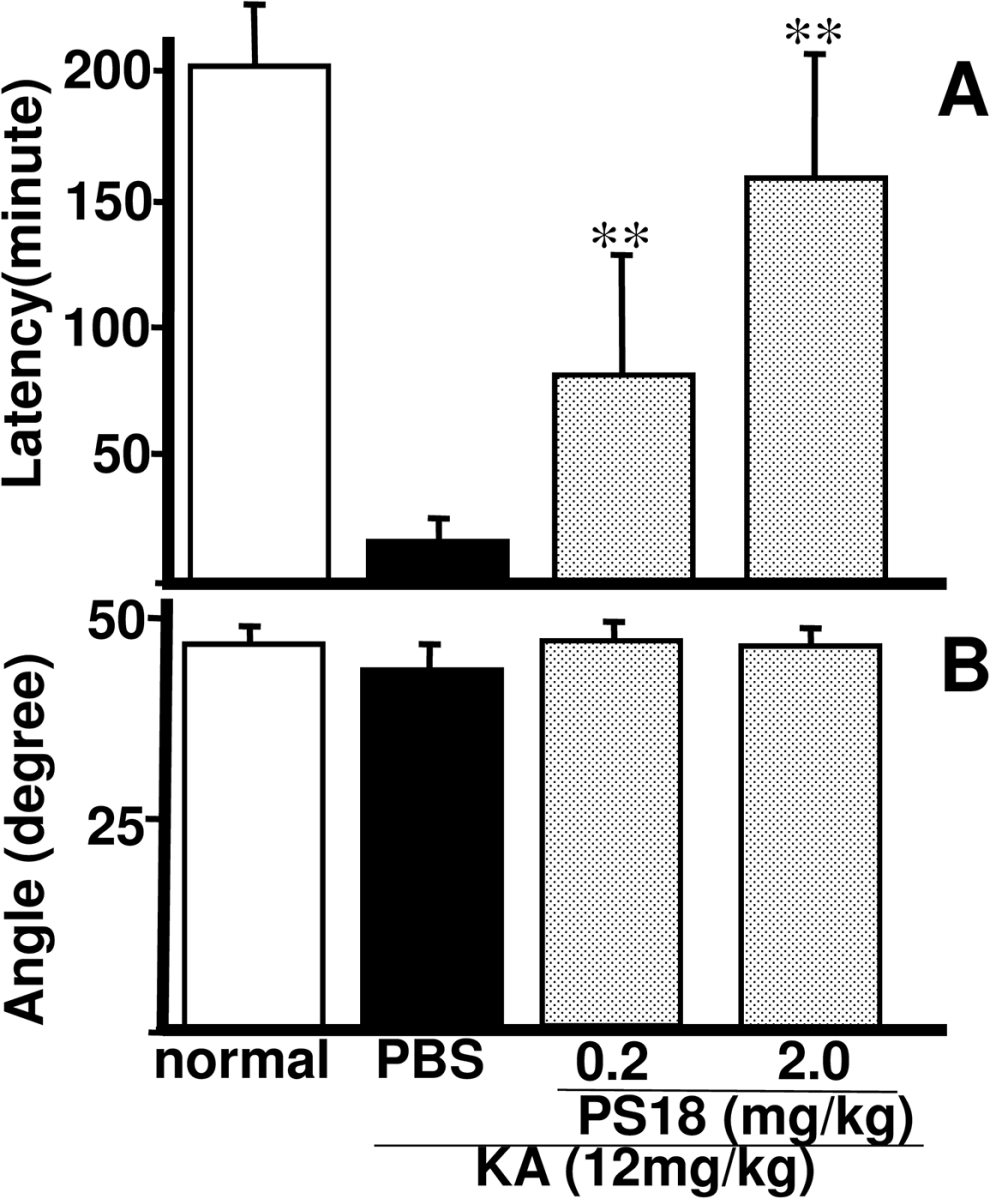
Passive Avoidance and Inclined Screen Tests. Effects of PS18 administration on learning disability (A) and the inclined screen test (B) in rats that received a subcutaneous injection of 12 mg/kg kainic acid (KA). Four PS18 treatments after a KA injection significantly prolonged response latency time in the passive avoidance task (A) in a dose-dependent manner in KA-injected rats compared with the PBS treatment after KA injection. *P < 0.05, **P < 0.01. A P-value < 0.05 was considered to be statistically significant. All data are expressed as mean ± standard error of the mean (S.E.M.).

Four PS18 injections following a KA injection caused a significantly prolonged response latency in the step-down passive avoidance task (0.2 and 2.0 mg/kg PS18 vs. PBS in KA-treated animals, *P* < 0.01; Fig 4A). The mean response latency of rats injected with 2.0 mg/kg PS18 after a KA injection was close to that of PBS-treated normal rats (2.0 mg/kg PS18-treated animals, 156.4 ± 53.4 s; PBS-treated normal rats, 205.6 ± 34.6 s; *P* <0.01).

In the present study, the angle of the inclined screen test did not significantly decrease in any group; however, the angle decreased by more than 5% in some rats injected with KA and PBS (Fig 4B).

### Histological Examination of KA-Induced Brain Injury

Light micrographs of the H&E-stained forebrain from PBS-treated normal rats (Fig 3A–3H), and that from KA and PBS-treated rats with the most severe neuronal damage (Fig 3I–3O) are shown. In the hippocampus of the latter rats, CA1, CA2, CA3, and CA4 neurons were severely damaged (Fig 3J–3M), but damage to the DG was less severe (Fig 3L). Many neurons were impaired in all layers of the piriform cortex (Fig 2P), but few neurons were damaged in other cortical regions (Fig 3N and 3O). The amygdaloid complex was also severely damaged (Fig 3I).

Using toluidine blue-stained hippocampal sections, we plotted injured neurons in rats that were injected with PBS (Fig 2B), 0.2 mg/kg PS18 (Fig 2C), or 2.0 mg/kg PS18 after a KA injection (Fig 2D). Our results demonstrated that KA-induced neuronal damage was intense in CA1 and CA4 (Fig 2B and 2C), but very weak in the DG (Fig 2B). Notably, CA3 damage was variable in some rats regardless of the total injury (Fig 2C and 2D); i.e., no CA3 neuron was damaged when the overall damage was moderate (Fig 2C), but many CA3 neurons were impaired when the overall damage was weak (Fig 2D). Exceptionally inverted cases (Fig 2D) were clearly observed in one rat, but cases with some damaged CA3 neurons occurred regardless of whether CA1 neurons were damaged.

### Light Microscopic Analysis of Hippocampal CA1

**Fig 5:**
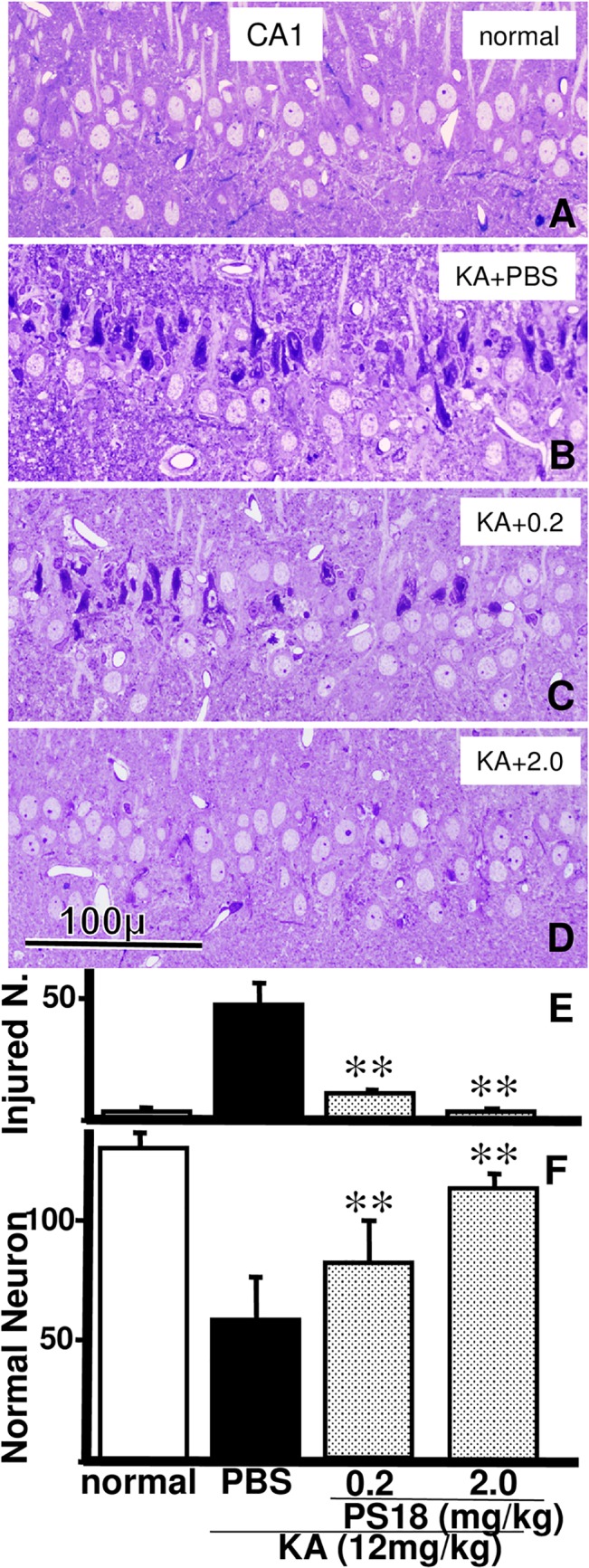
Light Microscopic Analysis of Hippocampal CA1. Photomicrographs of toluidine blue-stained hippocampal CA1 neurons in a normal control rat (A) and rats that received an injection of PBS (B), 0.2 mg/kg PS18 (C), or 2.0 mg/kg PS18 after a KA injection (D). Injured CA1 neurons were rescued by a PS18 treatment (C, D). Note that many injured neurons lined the apical side of the pyramidal layer. Scale bar = 100 μm. Effects of PS18 on injured (E) and viable (F) neurons in the hippocampal CA1 region in rats that received a subcutaneous injection of 12 mg/kg KA. PS18 treatment administered to KA-injected rats decreased the number of injured neurons (E) and increased the number of viable neurons (F) in a dose-dependent manner compared with the PBS-treated KA-injected rats. **P < 0.01. A P-value < 0.05 was considered to be statistically significant. All data are expressed as mean ± standard error of the mean (S.E.M.).

We counted injured and viable neurons in semithin toluidine blue-stained hippocampal CA1 sections harvested from rats injected with PBS (Fig 5B), 0.2 mg/kg PS18 (Fig 5C), or 2.0 mg/kg PS18 after a KA injection (Fig 5D). More injured neurons were observed in rats that received a PBS injection after the KA injection than rats that received a 0.2 or 2.0 mg/kg PS18 injection (Fig 5E). The mean number of viable CA1 neurons in normal rats was 132.4 ± 1.8 cells/mm, while that of KA-treated rats receiving PBS alone was significantly different (56.4 ± 12.2 cells/mm; *P* < 0.01); Fig 5F). Moreover, injured CA1 neurons showed a consistent tendency to line the apical side of the pyramidal cell layer (Figs 2B and 2C and 5B and 5C). Thus, a histological examination revealed that the PS18 treatment rescued many CA1 neurons that were destined to degenerate in the absence of the treatment.

### Electron microscopic Analysis of Hippocampal CA1

**Fig 6:**
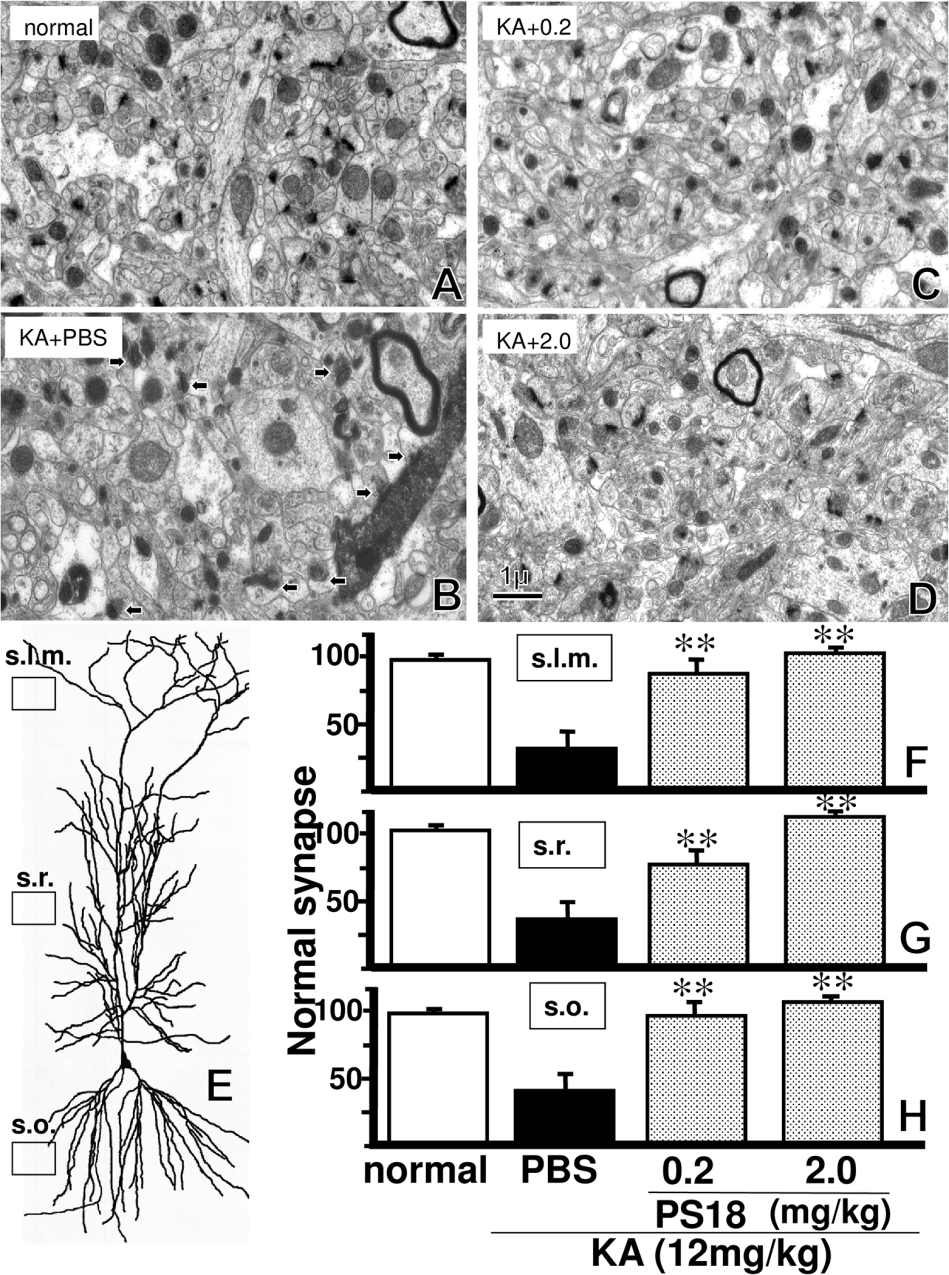
Electron microscopic Analysis of Hippocampal CA1. Electron micrographs of the hippocampal CA1 region stratum radiatum. A normal control rat that received a PBS injection without a kainic acid (KA) injection (A). Rat that received a PBS injection after a KA injection (B). Rat that received a PS18 injection (0.2 mg/kg) after a KA injection (C). Rat that received a PS18 injection (2.0 mg/kg) after a KA injection (D). Arrows indicate degenerated synapses. Note that the high electron density degenerated synapses were decreased in number by the PS18 injection. Scale bar = 1 μm. The rectangles in (E) indicate the areas where synapses were counted. A CA1 pyramidal neuron was re-created from Ishizuka et al [41] to illustrate the dendritic arborization of pyramidal neurons. The effect of PS18 on the number of normal synapses per 280 μm2 of the three strata of the hippocampal CA1 region is shown (F–H). Normal synapse number in the stratum lacunosum-moleculare (s.l.m; F), radiatum (s.r.; G), and oriens (s.o.; H) in the hippocampal CA1 region of KA-injected rats that received the PS18 treatment were more numerous than those in the respective strata of KA-injected rats that received the PBS treatment. **P < 0.01. A P-value < 0.05 was considered to be statistically significant. All data are expressed as mean ± standard error of the mean (S.E.M.).

Using electron microscopy, we determined that the stratum lacunosum-moleculare, radiatum, and oriens of the hippocampal CA1 region in PBS-treated normal rats contained 98.8±4.2, 102.2±5.1, and 96.7±4.5 synapses per 280 *μ*m^2^, respectively, while the CA1 of PBS-treated KA-injected rats respectively contained 41.7±12.2, 37.2±10.8, and 38.0±10.6 synapses per 280 *μ*m^2^ (Fig 6). Many degenerated postsynaptic structures and normal presynaptic structures were observed in the hippocampal CA1 region in rats receiving a PBS injection after KA injection (Fig 6B). The electron microscopy results showed that intact synapses within each stratum of the hippocampal CA1 region were more numerous in PS18-treated rats than in PBS-treated rats after the KA injection (Fig 6F–6H).

### Light Microscopic Analysis of Other Hippocampal Regions

**Fig 7:**
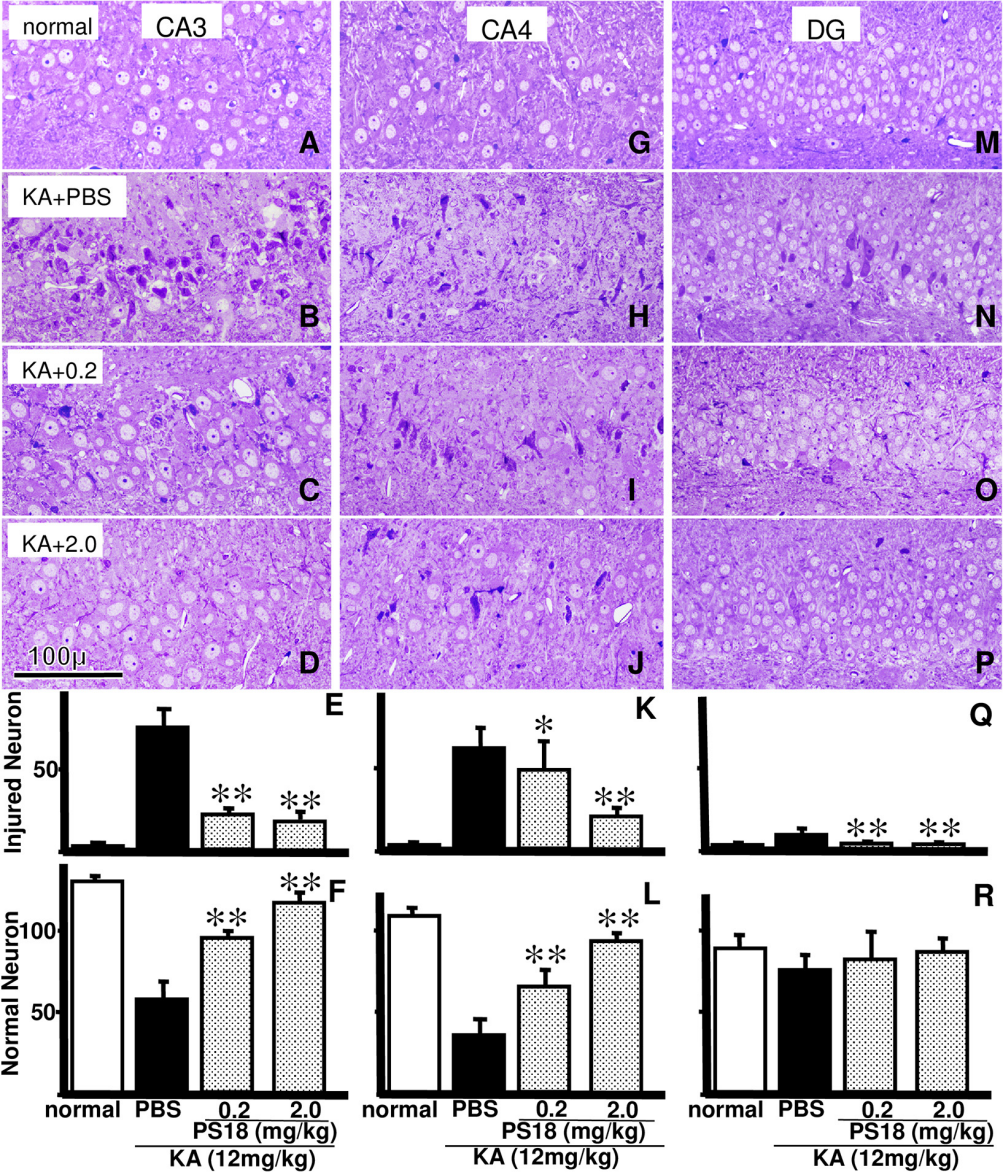
Light Microscopic Analysis of Other Hippocampal Regions. Photomicrographs of toluidine blue-stained hippocampal neurons in CA3 (A-D), CA4 (G–J), and the upper blade of the dentate gyrus (DG; M–P) in normal control rats (A, G, M) and rats that received an injection of PBS (B, H, N), 0.2 mg/kg PS18 (C, I, O), or 2.0 mg/kg PS18 after a KA injection (D, J, P). Injured CA1 neurons were rescued by the PS18 treatment. Note that some neurons were also injured in the DG (N). Scale bar = 100 μm. Effects of PS18 on injured (E, K, Q) and normal (F, L, R) neuronal density of the hippocampal CA1 region in rats that received a subcutaneous injection of 12 mg/kg kainic acid (KA). PS18 treatment following a KA injection in rats decreased the number of injured neurons (E, K, Q) and increased the number of viable neurons (F, L, R) in a dose-dependent manner compared with PBS-treated KA-injected rats. *P < 0.05, **P < 0.01. A P-value < 0.05 was considered to be statistically significant. All data are expressed as mean ± standard error of the mean (S.E.M.).

We counted injured and intact neurons in semithin hippocampal CA3, CA4, and DG sections stained with toluidine blue in rats that received injections of PBS (Fig 7B,7H and 7N), 0.2 mg/kg PS18 (Fig 7C,7I and 7O), or 2.0 mg/kg PS18 after a KA injection (Fig 7D,7J and 7P). Injured neurons in these three regions in rats that received a 0.2 or 2.0 mg/kg PS18 injection after the KA injection were fewer than those in rats receiving a PBS injection after the KA injection (Fig 7E,7K and 7Q). Viable CA1 neurons in the CA3 and CA4 regions of rats that received a 0.2 or 2.0 mg/kg PS18 injection after a KA injection were much greater than those in rats that received a saline injection after the KA injection (Fig 7F,7L and 7R). However, the number of viable neurons was similar in the DG of the three groups that received PBS, or 0.2 or 2.0 mg/kg PS18 (Fig 7R), regardless of the 9% decrease in viable neurons in rats that received a PBS injection after the KA injection compared to rats receiving a 2.0 mg/kg PS18 injection after the KA injection (Fig 7R).

### Light Microscopic Analysis of the Cerebral Cortex

**Fig 8:**
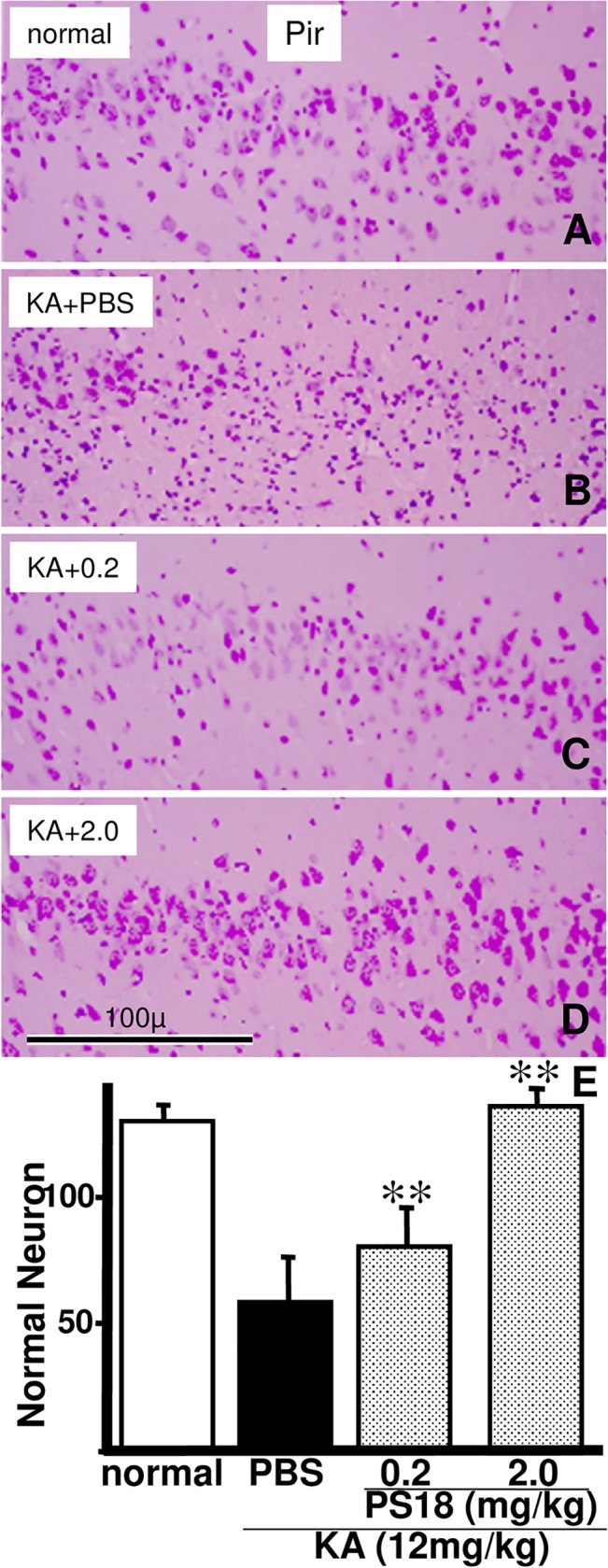
Light Microscopic Analysis of the Cerebral Cortex. Photomicrographs of hematoxylin and eosin (H&E)-stained neurons in the piriform cortex of a normal control rat (A) and rats that received injections of PBS (B), 0.2 mg/kg PS18 (C), or 2.0 mg/kg PS18 after a kainic acid (KA) injection (D). Injured neurons in layer II were rescued by the PS18 treatment (C, D). Scale bar = 100 μm. Effects of the PS18 treatment on viable neuronal density in the hippocampal CA1 region in rats that received a subcutaneous injection of 12 mg/kg KA (E). Four PS18 treatments following a KA injection decreased the number of injured neurons in a dose-dependent manner in KA-injected rats compared with PBS-treated KA-injected rats. **P < 0.01. A P-value < 0.05 was considered to be statistically significant. All data are expressed as mean ± standard error of the mean (S.E.M.).

Histological evaluation of the effects of KA and PS18 on the cerebral cortex was more difficult than that of the hippocampus because its histological structures are more complicated (Fig 8). Careful counting of viable pyramidal neurons in cerebral cortex layer V revealed no significant differences, although some damaged neurons were observed in this layer in rats that received a KA injection (Fig 3N and 3O). Viable piriform neurons in rats treated with 2.0 mg/day PS18 increased to reach a level similar to that of normal rats (2.0 mg/day PS18-treated rats, 141.2 cells/mm; PBS-treated normal rats, 136.8 cells/mm).

## Discussion

### Light Microscopic Analysis

In the present study, we used two light microscopy methods: paraffin sections stained with H&E (Figs 3 and 8) and semithin sections stained with toluidine blue (Figs 2,5 and 7). To examine the whole brain (Fig 3), we used H&E-stained ordinal paraffin sections, as H&E stains viable neurons well, but not injured neurons. To evaluate neuronal damage, the number of viable neurons is more important than the number of injured neurons; in this sense, H&E staining is also effective. However, in areas that contained few injured neurons (e.g., DG), sections stained with toluidine blue were more reliable for use in counting injured neurons as well as intact ones (Fig 7Q) because injured neurons were very darkly stained (Figs 2,5 and 7). In fact, the neuroprotective effect of PS18 in the DG was demonstrated only when the injured neurons were counted (Fig 7Q), but not when the viable neurons were counted (Fig 7R). Furthermore, with this method, the relative position of injured and viable neurons was evident because we were able to clearly observe both (Figs 5 and 7), which allowed us to demonstrate that the CA1 neurons on the apical side of the pyramidal cell layer were more vulnerable than those on the basal side (Fig 5B and 5C). This hard epoxy resin method is typically used for electron microscopy, and is limitation of small cutting area of glass or sapphire knifes. Regardless, toluidine blue-stained sections are very useful and reliable in evaluating cellular injury or amelioration effects as described above.

### KA Neurotoxicity

KA is a glutamate analog that stimulates excitatory neurotransmitter release, and a systemic KA injection induces neuronal degeneration in certain brain areas. The nature of KA-induced neuronal degeneration reportedly resembles some forms of ischemia [34]. However, neuronal damage following 12 mg/kg KA was much wider than that induced by 5 minutes ischemia in Mongolian gerbils, in which only hippocampal CA1 neurons were damaged [39,40].

Plotting of injured neurons (Fig 2) demonstrated that KA-induced neuronal damage was intense in CA1 and CA4 (Fig 2B and 2C), variable in CA3 (Fig 2B–2D), and very weak in the DG (Fig 2B). Although a statistical analysis revealed that KA-induced neuronal damage was ameliorated in a dose-dependent manner by PS18 injection in these hippocampal regions (Figs 5 and 7), the variation in CA3 is noteworthy, irrespective of exceptional cases (Fig 2C and 2D). The investigation into this cause is beyond the scope of this study, but some speculations are possible. For example, CA3 is a specific region where long-term potentiation (LTP) has been reported, and presynaptic kainate receptors are widely accepted to play an important role in frequency facilitation and LTP [42]. Furthermore, the hippocampus, particularly the DG, is the region where c-Fos expression (i.e., neuronal activation) is moderately observed under normal conditions, and is strongly observed following acute and chronic social stress [43]. The degree of KA-induced neuronal injury is influenced by temperature, anesthesia, or anticonvulsant drugs [15]. These delicate and complex mechanisms may be responsible for the variation in KA neurotoxicity in the CA3.

The neurons on the apical side of CA1 and CA3 were more damaged than those on the basal side (Figs 5B and 5C and 7B and 7C). This difference was not observed in ischemia, including in our own reports [39,40], which may be due to differences in excitatory inputs; many more excitatory inputs enter into the apical side of CA1 and CA3 [44]. Furthermore, the concentration of excitatory amino acids in the center of the hippocampus may be higher than that in the outer area. In line with this speculation, in the CA4 region, neuronal damage was more severe near the center of the hippocampus (Fig 7J); specifically, the upper blade of the DG in the center of the hippocampus (Fig 3B) contained injured neurons, but the lower blade did not (Fig 7N).

As reported by Kirino [42], many degenerated postsynaptic structures and normal presynaptic structures were observed in the hippocampal CA1 region in rats receiving PBS after KA injection (Fig 6B). Intact synapses within the hippocampus were more numerous in PS18-treated rats than in PBS-treated rats (Fig 6F–6H), and this result corresponded closely with results of the passive avoidance task (Fig 4A), and light microscopic observations (Fig 5F).

### Passive Avoidance and Inclined Screen Tests

Compared with the neuronal damage in CA1 (Fig 5F), the decrease in passive avoidance latency after KA injection was very severe (Fig 4A). In ischemia, the degrees of decreased neuronal damage and passive avoidance latency were similar: both were about 50–60% of that in control animals in Mongolian gerbils [39,40]. Conversely, in the present study, the latency decreased to 20%, irrespective of the 50% neuronal decrease (Figs 4A and 5F). The angle of the inclined screen test did not significantly decrease in any group; however, the angle decreased by more than 5% in some rats injected with KA and PBS, suggesting that muscle tone or coordinated four-leg movement was affected by the KA injection (Fig 4B). The inclined screen angle did not decrease even following severe ischemia-induced CA1 damage [39,40]. These differences may not be due to animal differences, but differences in the damaged areas. Only CA1 neurons were damaged after temporal ischemia; conversely, wide areas of the hippocampus (Figs 2 and 7) and cortex (Fig 8) were damaged after KA injection, and thus muscle tone or coordinated four-leg movement may have also been affected by the KA injection (Fig 4B).

### PS Receptors and Internalization

PS triggers a signal cascade after binding to G-protein-coupled receptor (GPR)37 or GPR37L1 [35], and these receptors are expressed in the cortex, hippocampus, cerebellum, and choroid plexus [38]. In lysosomes, PS is proteolytically processed to generate four sphingolipid activator proteins known as saposins A–D. In our experiments, fluorescence conjugated PS18 localized within lysosomes [26]. Thus, these receptors may uptake PS18, which then enters into cells and is absorbed by lysosomes where it might be hydrolyzed or transformed into other forms.

By tracing the movement of PS18-FAM in SH-SY5Y cells, we found that PS18 could enter the cell and be discharged 1 hour later, and that the retention time of PS18-FAM in the cytoplasm of 1-methyl-4-phenylpyridinium (MPP+)-treated cells was shorter than that in untreated cells [26], which suggests that the biological activity of PS18 is related to its metabolism. A specific 18-amino-acid peptide prevented nerve damage and associated cognitive impairment in both gerbil and rat ischemic models when administered via intracerebroventricular injection [24,45]. This peptide appears to ameliorate ischemic brain damage and may not be degraded or may be more slowly degraded in the central nervous system (CNS).

### Neurotrophic Activity of PS18

*In vivo* experiments demonstrated that a PS or PS-peptide treatment protected neurons from focal cerebral ischemia in rats, and prevented ischemia-induced learning disability and hippocampal CA1 neuronal loss in gerbils [23,24]. PS and synthetic peptides promote survival and neurite outgrowth *in vitro* [17–20,22,24,46]. These *in vivo* and *in vitro* findings suggest that the biological activity of PS18 produces effects similar to that of intact PS; PS prevented cellular apoptosis [47] through extracellular signal-regulated kinase (ERK) phosphorylation and sphingosine kinase [48]. PS-peptide also increased mitogen-activated protein kinase (MAPK) phosphorylation in rat pheochromocytoma (PC-12) cells [49] and promotes Schwann cell survival via the ERK and phosphatidylinositol-3-kinase (PI3K)–Akt pathway [20,50]. Under oxidative stress, it prevented the death of PC-12 cells via ERK phosphorylation and the inhibition of Akt, c-Jun N-terminal kinase (JNK), and p38 phosphorylation [51]. *In vitro*, PS18 also (1) protected SH-SY5Y cells from MPP^+^-induced cell damage via suppression of the JNK/c-Jun pathway, (2) upregulated Bcl-2, (3) downregulated BAX, attenuating mitochondrial damage, and (4) inhibited caspase-3 [26].

In summary, the present study indicated that PS18 exerts neuroprotective effects against KA-induced neuronal death *in vivo* and suggests that subcutaneously-injected PS-derived peptides work consistently, without rapid degradation in the body, and ameliorate nerve dysfunction. Our data provide experimental evidence regarding the mechanism of the neuroprotective function of PS and allow for exploration of the pharmaceutical use of PS18 as an approach to the prevention and treatment of neuronal injury.

For the replacement, refinement or reduction (3Rs) of the use of animals in research, all animals were used for both electron and light microscopy after the passive avoidance and inclined screen tests (following anesthetiesia with diethyl ether). The moderate dose of KA after clonazepam treatment was not lethal and did not induce convulsions.
